# Enhancement of the Humidity Sensing Performance in Mg-Doped Hexagonal ZnO Microspheres at Room Temperature

**DOI:** 10.3390/s19030519

**Published:** 2019-01-26

**Authors:** Cunchong Lin, Hongyan Zhang, Jun Zhang, Chu Chen

**Affiliations:** School of Physical Science and Technology, Xinjiang University, Urumqi 830046, China; lccz1213@163.com (C.L.); chenchu@xju.edu.cn (C.C.)

**Keywords:** sol-gel, black ZnO, Mg-doped ZnO, relative humidity sensors

## Abstract

In this paper, Mg-doped black ZnO microspheres with the characteristics of large surface area and surface oxygen vacancies were synthesized using the sol-gel method. The humidity sensing behavior of the Mg-doped ZnO for relative humidity (RH) from 11% to 95% was measured at room temperature. The superior humidity sensing performance recorded for Mg-doped ZnO microspheres (1.5 mol%) exhibits a dramatic change of impedance of about four orders of magnitude, excellent sensing linearity, small hysteresis (4.1%), a fast sensing response time of as low as 24 s, and a recovery time of 12 s. Our studies demonstrate that Mg dopant can significantly enhance the humidity sensing performance of black ZnO. This benefits from the Mg-doped ZnO (1.5 mol%) microspheres having a high level of surface adsorption and the abundant oxygen vacancies on the surface. Such a new material could be very useful to develop simple and high-performance humidity sensors for future applications in varying commercial fields and industries.

## 1. Introduction

Humidity, the content of water vapor in the atmosphere, has a great impact on our lives, including agriculture, living environment, food storage, and industrial production. It is necessary to monitor and control the humidity to increase human comfort and product quality [1,2,3,4]. Measuring humidity is essential in several areas, including the environment, clinical practice, agriculture, food, industry, and biotechnology. This highlights the importance of developing all kinds of humidity sensors with simple fabrication, high sensitivity, and low cost [5]. Modern humidity sensing most commonly measures the change in resistance of a device to determine differences in humidity [6,7]. Polymers, composites, ceramics, and metal oxide semiconductors are common functional materials for resistive-type humidity sensors [8,9,10]. For the above materials, the advantages of metal oxide semiconductors lie in their rich resources, controllable size and morphology, simple synthesis, and good physical and chemical stability [11]. ZnO is a versatile II–VI oxide semiconductor with a wide band gap (3.37 eV) and a hexagonal wurtzite structure. It exhibits interesting properties in transducers, lasers, biosensors, optoelectronic gas, and humidity detection [12,13]. Additionally, ZnO is believed to be contained in nontoxic materials in our daily lives [14]. Some literature has reported that ZnO can be doped successfully to prepare a black semiconductor humidity sensitive material, which has better humidity sensing performance than traditional semiconductor materials [15,16]. However, there has been limited research into black ZnO and its influence on relative humidity (RH) sensing performance as far as we know [17]. In order to widen the range of ZnO materials in humidity sensors, we have chosen black ZnO as the research system of our humidity sensor.

We know that humidity sensing performance is influenced by grain shape, surface area to volume ratio, and a surface defect of crystalline [18,19]. Therefore, many researchers have changed the structure and shape of ZnO by selecting suitable elements as dopants, thus obtaining high performance moisture sensitive sensing materials [20,21]. Among these different elements, Mg is considered to be able to potentially affect one of the sensing performance elements of ZnO. This is due to the high solubility of Mg in ZnO, excellent electrical conductivity, and its effective catalytic nature [22,23,24]. In addition, Mg^2+^ (0.57 Å) and Zn^2+^ (0.60 Å) have a similar ionic radius and Mg doping does not affect the ZnO lattice constant [25]. At present, research on Mg-doped ZnO-based semiconductor materials has achieved great results in promoting photocatalysis and optoelectronic applications [26,27,28]. However, research work on Mg-doped ZnO humidity sensors has rarely been reported. To this end, we will conduct in-depth research on the moisture sensitivity mechanism of Mg-doped ZnO-based semiconductor materials in order to develop an effective control method and try to manufacture a high-performance Mg-doped ZnO-based humidity sensor.

In this paper, Mg-doped black ZnO microspheres were fabricated by sol-gel and were then used to detect humidity at room temperature. It was found that the humidity sensor based on Mg-doped ZnO microspheres (1.5 mol%) has a high sensitivity, fast response, good linearity, and low hysteresis. This means that Mg-doped ZnO microspheres (1.5 mol%) have the potential to enhance humidity sensing performance due to the large surface area, surface oxygen vacancies, and high electron mobility. Such a method is a useful way to achieve high-performance humidity sensors.

## 2. Materials and Methods

### 2.1. Materials

The raw materials of Zinc acetate (Zn(CH_3_COO)_2_·2H_2_O), monoethanolamine (MEA), and magnesium chloride hexahydrate (MgCl_2_·6H_2_O) were purchased from Macklin (Shanghai, China) and directly used without further purification. Deionized water and absolute ethanol were used in the whole experiment.

### 2.2. Apparatus

The structural characteristics of samples were analyzed through the X-ray diffraction (XRD) (Bruker, Karlsruhe, Germany) patterns (2θ = 15–90°, rate of 6 deg min^−1^) from a Bruker D8 Advance X-ray Diffraction using Cu Kα radiation. A field emission scanning electron microscope (SEM) (Hitachi, Japan) was used to characterize the morphology of the samples. The chemical bonding state of samples was analyzed by X-ray photoelectron spectroscopy (XPS) (Bruker, Kronach, Germany) using a Thermo VG ESCA Lab250 spectrometer. Absorption spectra with a wavelength of 200.00 to 1000.00 nm were obtained by a Lambda 650 UV-Vis spectrophotometer (Perkin Elmer, Karlsruhe, Germany) with a gold coating. The impedance of humidity sensors was measured by a Zennium workstation (Zahner, Kronach, Germany).

### 2.3. Synthesis of Mg-Doped ZnO Microspheres

In this experiment, 2.3 g Zn(CH_3_COO)_2_·2H_2_O and a certain amount of MgCl_2_·6H_2_O were dissolved in a mixed liquid of 20 mL ethanol and 10 mL deionized water. The molar ratio of Mg^2+^ ions ranged from 0 mol% to 2 mol% after being stirred for 5 minutes in a 65 °C water bath. One milliliter of ethanolamine (MEA) was slowly dripped into the stirred in a 65 °C water bath for 2 h to get white uniform sol. After being aged at room temperature for 36 h, all aged sols were calcined in a quartz tube furnace under nitrogen (N_2_) environmental protection for 2 h.

### 2.4. Preparation of Humidity Sensors

**Figure 1:**
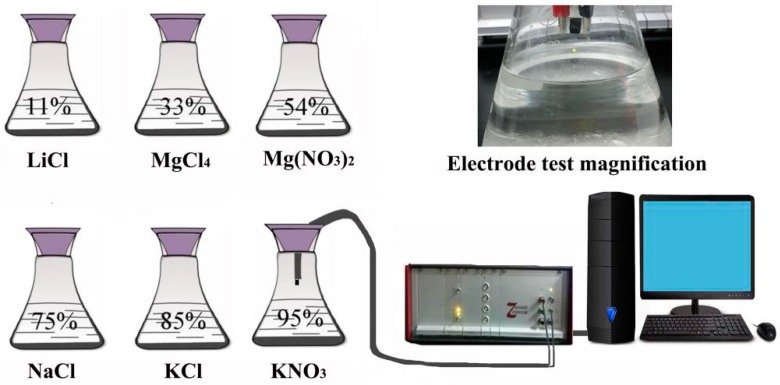
The diagram of humidity sensor measurement system.

At first, the DI water and the synthesized samples were thoroughly mixed in a weight ratio of 3:1 and ground to a diluted paste. Secondly, in order to form a good sensitive film, the paste was uniformly applied on the ceramic substrate (40 mm × 70 mm) of five double-crossed Ag-Pd electrodes with a small brush, then aged for 48 h. Finally, the humidity sensitivity of the prepared thin film electrodes was tested at 11%, 33%, 54%, 75%, 85%, and 95% RH. These humidity environments were controlled by supersaturated aqueous salts solutions of LiCl, MgCl_2_, Mg(NO_3_)_2_, NaCl, KCl, and KNO_3_ in a closed glass containers. In order to measure the resistance characteristic curve of the Mg-doped ZnO humidity sensor changing with RH, the voltage of the Zennium workstation was always kept at AC 1 V during the whole experiment, and the measurement frequency ranged from 40 Hz to 100 kHz. The humidity sensor measurement system can be found in Figure 1.

## 3. Results and Discussion

**Figure 2:**
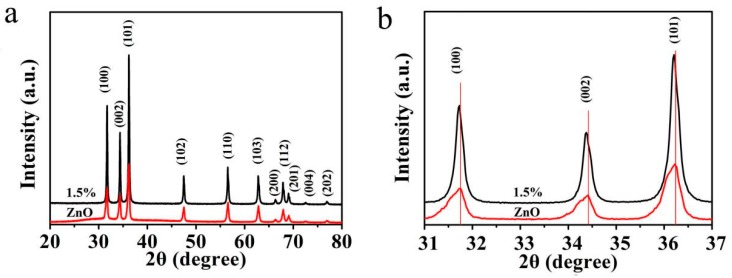
(**a**) XRD patterns of undoped ZnO and Mg-doped ZnO (1.5 mol%). (**b**) Magnified XRD patterns.

XRD patterns of the undoped ZnO and Mg-doped ZnO (1.5 mol%) are shown in Figure 2.

Figure 2a shows that the diffraction peaks of the synthesized undoped ZnO and Mg-doped ZnO samples correspond to (100), (002), (101), (102), (110), (103), (200), (112), (201), (004), and (202). All the diffraction peaks point to the formation of ZnO hexagonal wurtzite structure (JCPDS No. 80-0075). There are no characteristic peaks of MgO and other impurities in the XRD pattern, indicating that the Mg^2+^ ions successfully replaced the Zn^2+^ ions in the ZnO lattice [29,30]. Compared with undoped ZnO, Mg-doped ZnO samples showed a stronger diffraction peak, indicating that the growth of nanoparticles forming Mg-doped ZnO (1.5 mol%) microspheres showed a high priority orientation. In Figure 2b, there is a small offset of diffraction peaks to lower angles for Mg-doped ZnO, which might be due to the Mg^2+^ dopant causing distortion of the lattice of ZnO and generation of crystal defects around the dopants [31]. The results show that the microspheres will have more active sites, which enhances the adsorption characteristics of the microspheres on water molecules.

**Figure 3:**
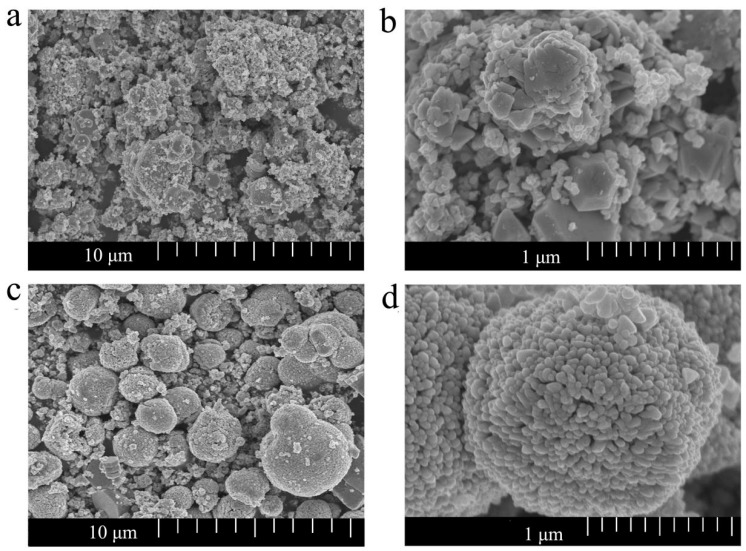
SEM images of (**a**,**b**) undoped ZnO and (**c**,**d**) Mg-doped ZnO (1.5 mol%) nanostructures at different multiple resolutions.

Figure 3a,b display the microstructure of undoped ZnO at different resolutions. It turns out that the undoped ZnO possesses irregular massive particles and the size is from 0.1 μm to 0.4 μm. Figure 3c shows that Mg-doped ZnO (1.5 mol%) samples consist of a large number of regular microspheres with diameters varying from 0.1 μm to 0.4 μm. As shown in Figure 3d, the high-magnification SEM images of ZnO show that the microspheres are assembled by thousands of nanoparticles with an average size of about 10 nm. This means that the Mg-doped ZnO nanostructured microspheres are aggregated by uniform size nanoparticles. Such a structure undoubtedly increases the surface roughness and specific surface area. These results showed that Mg^2+^ doping promoted the great changes of ZnO nanostructure [32]. Thus, a large roughness surface area, good stability, and high activity Mg-doped ZnO microspheres were obtained. 

**Figure 4:**
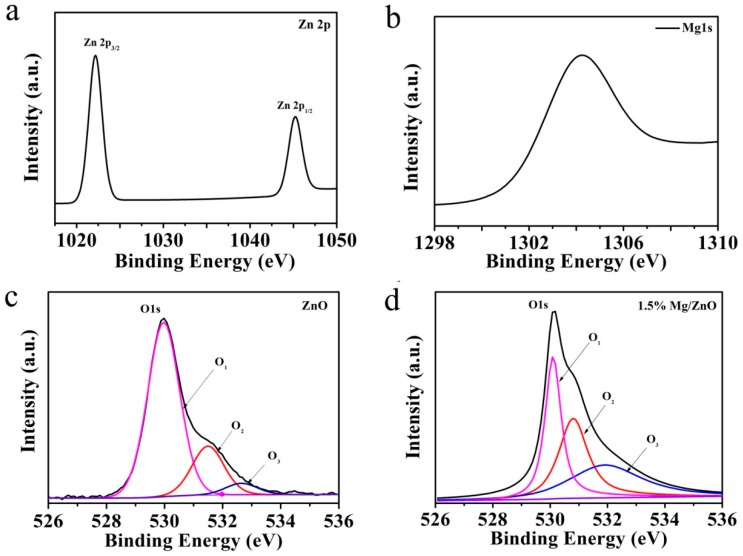
The XPS spectra of (**a**) Zn 2p, (**b**) Mg (1 s) of Mg-doped ZnO (1.5 mol%). The XPS spectra of O (1 s) of (**c**) undoped and (**d**) Mg-doped ZnO (1.5 mol%), respectively.

The X-ray photoelectron spectroscopy (XPS) technology was used to analyze the chemical states of the elements of Mg-doped ZnO in Figure 4. The peaks from Zn 2p, Mg 1s and O 1s can be clearly observed without any other impurity peaks. Figure 4a shows the Zn 2p consists of two peaks 1021.6 eV and 1044.7 eV corresponding to Zn 2p_3/2_ and Zn 2p_1/2_, respectively. Additionally, 23.1 eV peak separation corresponds to the standard reference value of ZnO, indicating that the Zn atom is in a +2 oxidation state [33]. From Figure 4b, it can be obtained that the bonding energy peak for Mg 1s is 1304.2 eV, and Mg can be expected to be in the +2 oxidation state [34]. In Figure 4c,d, the O 1s peak of the high-resolution spectrum were Gaussian fitted into three peaks of O_1_, O_2_, and O_3_. The highest banding energy peak of O_1_ is centered at around 530 eV, which can be attributed to the O^2−^ ion in the hexagonal ZnO structure. The O_2_ peak intensity at 531.5 eV represents the oxygen vacancy defect concentration. The peak O_3_ of the binding energy 532.7 eV can be attributed to hydroxyl species presented on the surface of the ZnO [35]. The O_2_ peak intensity of Mg-doped ZnO has a higher peak intensity than the undoped ZnO, and the O_2_ peak area ratio changes from 14.19% t to 45.15% t, indicating that the dopant greatly increases the oxygen vacancy concentration. This result is an increase in oxygen vacancies due to the difference in electronegativity and ionic radius between Zn and Mg during the substitution of Zn^2+^ by Mg^2+^ in the ZnO lattice [36]. Some previous studies have shown that oxygen vacancies on the surface increase the concentration of the charge carriers and are therefore able to improve the humidity sensing performance [37]. Consequently, Mg-doped ZnO (1.5 mol%) microspheres with abundant oxygen vacancy have excellent humidity sensitivity.

**Figure 5:**
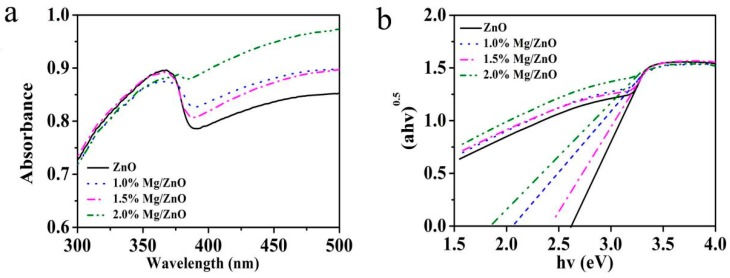
UV-Vis absorption spectra (**a**) and the band gap energy (**b**) of the Mg-doped ZnO with different Mg contents.

In Figure 5, the optical properties of different contents of Mg-doped ZnO were determined by UV-Vis diffuse reflectance spectra. It can be seen from the figure that the absorption of Mg-doped ZnO samples in the visible light region were significantly enhanced. Compared with pure ZnO, as the Mg^2+^ doping amount increases, the absorption band edge of the Mg-doped ZnO sample shifts toward the wavelength (red shift). This red shift behavior may be that Mg^2+^ was successfully doped into ZnO lattice, resulting in 3d–4s and 4d–5s orbital electron interactions and reduced electron density [38].

The band gap energies (Eg) of the sample are determined by Kubelka–Munk (F(R)) formula. F(R) can be derived from the relation F(R) = (1 − R)^2^/2R = K/S, where R, S and K represent the reflectivity, scattering and absorption coefficient, respectively. The band gap energies of the samples with Mg/Zn molar ratios of 0, 1.0%, 1.5%, and 2.0% can be calculated by plotting 2.65 eV, 2.05 eV, 2.40 eV and 1.80 eV. It is obvious that the band gap energy of all Mg-doped ZnO samples are significantly lower than that of undoped ZnO samples, which means that Mg^2+^ doping can reduce the band gap of ZnO samples.

**Figure 6:**
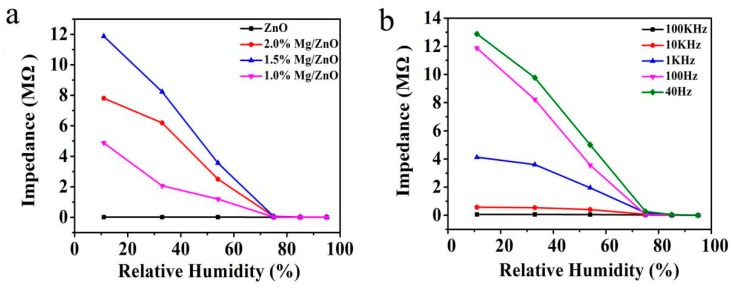
(**a**) Impedance versus RH curves of a Mg-doped ZnO humidity sensor with different ratios, (**b**) relationship of impedance and RH about Mg-doped ZnO (1.5 mol%) microspheres at various frequencies.

Figure 6a shows the dependence of impedance on the RH for ZnO sensors containing different contents of Mg^2+^. The test frequency and AC voltage were selected as 100 Hz and 1 V, respectively. As RH increases from 11% to 95%, the impedance of undoped ZnO decreases slightly and the impedance of Mg-doped ZnO decreases considerably more. Of all the above samples, the Mg-doped ZnO (1.5 mol%) sample had the best linearity and maximum impedance change (about 4 orders of magnitude). Therefore, it can be determined that the Mg-doped ZnO (1.5 mol%) sample has the best doping ratio of Mg ^2+^. It can be seen from the SEM that the surface of Mg-doped ZnO (1.5 mol%) microspheres are composed of highly uniform nanoparticles. Such a structure has a large roughness surface and a large number of active sites. As a result, more water molecules are easily attached to the ZnO microsphere surface. Considering all of the above discussions, Mg-doped ZnO (1.5 mol%) microspheres were selected for humidity performance measurements in the following experiments.

To determine the optimal measuring frequency, we tested the correspondence between RH and impedance at different frequencies, as shown in Figure 6b. It can be seen that at low frequencies (40 Hz and 100 Hz), the Mg-doped ZnO (1.5 mol%) humidity sensor has the best sensitivity and linearity. However, at high frequencies (1 kHz, 10 kHz and 100 kHz), the linearity between impedance and RH is very poor. In addition, the humidity sensor will become unstable when the operating frequency is too low. Considering the high response and stability of impedance under full RH range at 100 Hz frequency, we chose it to work at 100 Hz.

**Figure 7:**
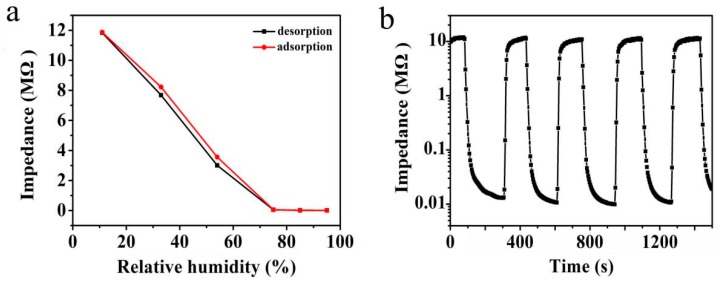
(**a**) Humidity hysteresis curve of Mg-doped ZnO microspheres, (**b**) response and recovery characteristic curve of Mg-doped ZnO microspheres (1.5 mol%).

Figure 7a illustrates the humidity hysteresis characteristics of the humidity sensor with Mg-doped ZnO microspheres (1.5 mol%) measured at 100 Hz. The red solid line in Figure 7a represents the adsorption process and the black solid line represents the desorption process. The sensor indicates that the desorption curve is slightly below that of the adsorption and that the two curves for the respective processes almost imitate each other. In this process, hysteresis error is determined by the following equation:

(1) γH=±ΔHmax2FFS

where Δ*H*_max_ is the output difference between adsorption and desorption process and *F_FS_* is the full scale output [16].

In our experiments, Mg-doped ZnO microspheres show an excellent hysteresis value of less than 4.1%. A narrow humidity hysteresis loop indicates a good reversible performance and slight immaterial hysteresis of this sensor.

In industrial production, response and recovery characteristics of humidity sensors are very important and have to be tested for such a humidity sensor. Figure 6b shows the response and recovery characteristic curve of Mg-doped ZnO (1.5 mol%) microspheres measured at 100 Hz with RH from 11% to 95% RH. Special attention is paid to the time required for the total resistance change of 90% as the measurement response and recovery time standard. In the figure, the time interval between two points is 2 s, it can be clearly seen that the response time is about 24 s (sensor moves from 11% to 95% RH), and the recovery time is about 12 s (sensor moves from 95% RH to 11% RH). The humidity sensor measures four basic similar curves in different test cycles, meaning that the humidity sensor has good repeatability. In addition, it can be observed that any curve has four orders of magnitude impedance variation. In our experiments, a four order of resistance change for humidity sensor of Mg-doped ZnO is similar to that of recently reported resistivity-type ZnO humidity sensor, such as Er-doped black ZnO nanocrystallines with three orders of magnitude [17], MoS_2_-modified ZnO quantum dot nanocomposites with three or four orders of magnitude [39], and ZnO dandelion-like structures with three orders of magnitude [40].

**Figure 8:**
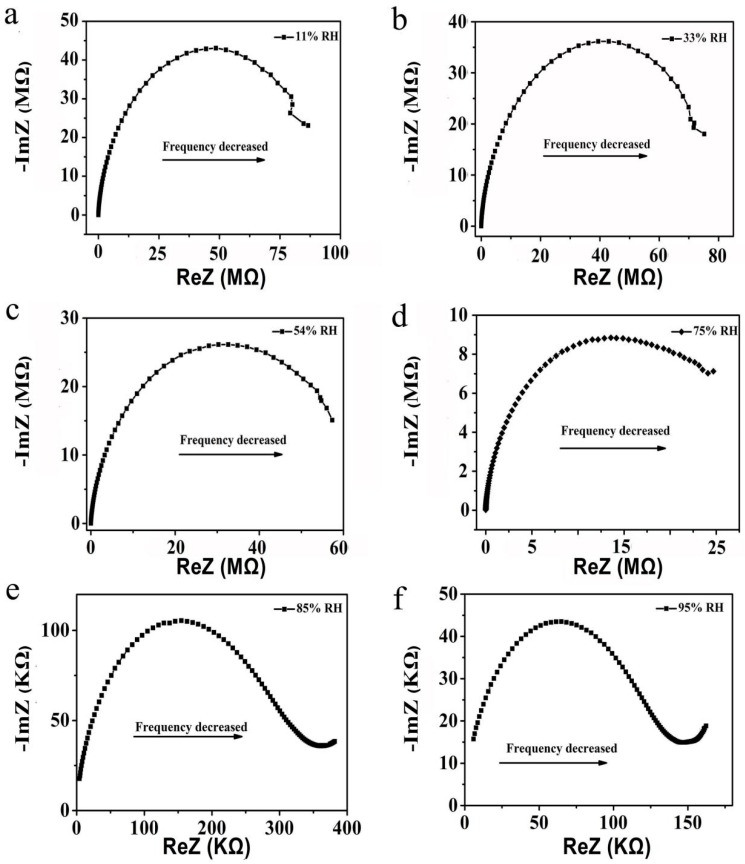
Complex impedance curves of Mg-doped ZnO microspheres (1.5 mol%) in (**a**) 11% RH, (**b**) 33% RH, (**c**) 54% RH, (**d**) 75% RH, (**e**) 85% RH and (**f**) 95% RH, respectively.

In order to study the humidity sensing mechanism, the complex impedance of the Mg-doped ZnO microspheres have been measured as shown in Figure 8. In low humidity (11%, 33%, 54% RH), the complex impedance curve appears semicircular. Kannan et al. reported that at relatively low humidity, chemisorption process occurs at ZnO surface defects, leading to the presence of negatively charged species such as O_2_^−^, O^−^, O^2−^ or OH^−^ [41,42]. In this experiment, XPS results proved that doping Mg^2+^ ions will cause rich surface oxygen vacancies on the surface of ZnO microspheres. Oxygen vacancies promoted the absorption of more water molecules on the surface of Mg-doped ZnO microspheres. The absorbed water molecules reacted with the surface oxygen vacancy defects to produce OH^−^ and H^+^. At this stage, abundant oxygen vacancy defects on the surface of ZnO are beneficial for hopping transportation of protons from site to site across the surface of the microspheres and the conduction is caused by protons in the low RH range. As a consequence, oxygen vacancy defects exhibit a high local electric field, promoting the dissociation of water molecules (Equation (2)). The equations can be expressed as follows [37]:

2H_2_O + O_v_ + O_b_ → O_v_–OH^−^ + O_b_–H^+^ (2)

where O_v_ is oxygen vacancy defects and O_b_ is bridging-oxygen.

With the increase of RH (75%, 85%, and 95% RH), physical adsorption of water molecules takes place, and one or more water layers are connected in series between ZnO microspheres, resulting in capillary condensation [4]. As a result, more H_3_O^+^ ions are able to transfer freely to promote the electron transport formation, in which H_3_O^+^ ions are dominant charged carriers and they accelerate the electron conduction by releasing protons into water molecules nearby. The quick transfer of H_3_O^+^ ions on the water layers results in a decrease of the impedance of Mg-doped ZnO microspheres. In Figure 8e,f, the end of the semicircle rises at a low frequency, and the upturned line becomes more and more obvious, meaning that more conduction ions move freely among the microspheres, increasing the electrical conduction of the sensors.

In the above humidity process, doping Mg^2+^ can offer unique physicochemical and electronic properties which will play a very important role in humidity sensors. In the case of low RH level on the surface of Mg-doped ZnO, abundant oxygen vacancies provide a highly local charge density and hence a strong electrostatic field. The adsorbed water molecules can then be decomposed into H^+^ and OH^−^ ions. In the case of high RH, the doping of Mg^2+^ induces a large number of adsorption sites on the surface of ZnO microspheres to enhance more water molecules to be absorbed, which results in a large number of H_3_O^+^ as dominant charged carriers. Therefore, the specific surface area will enhance humidity sensing performance of the Mg-doped ZnO microspheres.

## 4. Conclusions

To summarize, we synthesized Mg-doped black ZnO microspheres using the sol-gel method and investigated their structure, morphology, and humidity sensing performance. These results demonstrate that a highly sensitive humidity sensor has been discovered using Mg-doped black ZnO microspheres. In these samples, the Mg-doped ZnO (1.5 mol%) shows about four orders of magnitude for sensitivity, good linearity, low humidity hysteresis, and rapid response speed from 11% to 95% RH at room temperature. The recovery and the response times of Mg-doped black ZnO are 12 s and 24 s, respectively. The enhanced response to water molecules basically comes from the changes in resistance due to the large amount of adsorption sites and oxygen vacancies on the surface of Mg-doped ZnO microspheres, which can result in a high density of conductivity H_3_O^+^. Our results demonstrate that Mg^2+^ dopant into black ZnO growth is a very effective method to improve humidity sensing performance.

## References

[1] Wang W., Li Z., Liu L., Zhang H., Zheng W., Wang Y., Huang H., Wang Z., Wang C. (2009). Humidity sensor based on LiCl-doped ZnO electropunk nanofibers. Sens. Actuators B Chem..

[2] Qi Q., Zhang T., Yu Q., Wang R., Zeng Y., Liu L., Yang H. (2008). Properties of humidity sensing ZnO nanorods-base sensor fabricated by screen-printing. Sens. Actuators B Chem..

[3] Fei T., Jiang K., Liu S., Zhang T. (2014). Humidity sensors based on Li-loaded nanoporous polymers. Sens. Actuators B Chem..

[4] Tsai F.S., Wang S.J. (2014). Enhanced sensing performance of relative humidity sensors using laterally grown ZnO nanosheets. Sens. Actuators B.

[5] Toloman D., Popa A., Stan M., Socaci C., Biris A.R., Katona G., Tudorache F., Petrila I., Iacomi F. (2017). Reduced graphene oxide decorated with Fe doped SnO_2_ nanoparticles for humidity sensor. Appl. Surf. Sci..

[6] Zhu Y., Li Q., Wang P., Zang W., Xing L., Xue X. (2015). Enhanced piezo-humidity sensing of Sb-doped ZnO nanowire arrays as self-powered/active humidity sensor. Mater. Lett..

[7] Li Y., Hong L., Yang M. (2008). Crosslinked and quaternized poly (4-vinylpyridine)/polypyrrole composite as a potential candidate for the detection of low humidity. Talanta.

[8] Yuan Q., Li N., Tu J., Li X., Wang R., Zhang T., Shao C. (2010). Preparation and humidity sensitive property of mesoporous ZnO–SiO_2_ composite. Sens. Actuators B Chem..

[9] Zhang T., Wang R., Geng W., Li X., Qi Q., He Y., Wang S. (2008). Study on humidity sensing properties based on composite, materials of Li-doped mesoporous silica A-SBA-15. Sens. Actuators B Chem..

[10] Qi Q., Zhang T., Wang S., Zheng X. (2009). Humidity sensing properties of KCl-doped ZnO nanofibers with super-rapid response and recovery. Sens. Actuators B Chem..

[11] Zhao J., Liu Y., Li X., Lu G., You L., Liang X., Liu F., Zhang T., Du Y. (2013). Highly sensitive humidity sensor based on high surface area mesoporous LaFeO_3_ prepared by a nanocasting routen. Sens. Actuators B Chem..

[12] Turgut G., Duman S., Sonmez E., Ozcelik F.S. (2016). A study of Eu incorporated ZnO thin films: An application of Al/ZnO:Eu/p-Si heterojunction diode. Mater. Sci. Eng. B.

[13] Zhang Y., Liu Y., Wu L., Li H., Han L., Wang B., Xie E. (2009). Effect of annealing atmosphere on the photoluminescence of ZnO nanospheres. Appl. Surf. Sci..

[14] Khataee A.R., Karimi A., Soltani R.D.C., Safarpour M., Hanifehpour Y., Joo S.W. (2014). Europium-doped ZnO as a visible light responsive nanocatalyst: Sonochemical synthesis, characterization and response surface modeling of photocatalytic process. Appl. Catal. A Gen..

[15] Zhang H., Zhang M., Lin C., Zhang J. (2018). AuNPs Hybrid Black ZnO Nanorods Made by a Sol-Gel Method for Highly Sensitive Humidity Sensing. Sensors.

[16] Tomer V.K., Duhan S., Sharma A.K., Malik R., Nehra S.P. (2015). One pot synthesis of mesoporous ZnO-SiO_2_ nanocomposites as high performance humidity sensor. Colloids Surf. A Physicochem. Eng. Asp..

[17] Zhang M., Zhang H., Li L., Tuokedaerhan K., Jia Z. (2018). Er-enhanced humidity sensing performance in black ZnO-based sensor. J. Alloys Compd..

[18] Chang S.-P., Chang S.-J., Lu C.-Y., Li M.-J., Hsu C.-L., Chiou Y.-Z., Hsueh T.-J., Chen I.C. (2010). A ZnO nanowire-based humidity sensor. Superlattices Microstruct..

[19] Chen X., Liu L., Peter Y.Y., Mao S.S. (2011). Increasing solar absorption for photocatalysis with black hydrogenated titanium dioxide nanocrystals. Science.

[20] Zhu D., Hu T., Zhao Y., Zang W., Xing L., Xue X. (2015). High-performance self-powered/active humidity sensing of Fe-doped ZnO nanoarray nanogenerator. Sens. Actuators B Chem..

[21] Yu S., Zhang H., Lin C., Bian M. (2019). The enhancement of humidity sensing performance based on Eu-doped ZnO. Curr. Appl. Phys..

[22] Ohtomo A., Kawasaki M., Koida T., Masubuchi K., Koinuma H., Sakurai Y., Yoshida Y., Yasuda T., Segawa Y. (1998). Mg_x_Zn_1−x_O as II–VI widegap semiconductor alloy. Curr. Appl. Phys..

[23] Chen C., Yu W., Liu T., Cao S., Tsang Y. (2017). Graphene oxide/WS2/Mg-doped ZnO nanocomposites for solar-light catalytic and antibacterial applications. Sol. Energy Mater. Sol. Cells.

[24] Jia Y., Sun H., Liang H., Ji H., Song L., Gao C., Xu H. (2016). A highly efficient metal-free oxygen reduction electrocatalyst assembled from carbon nanotubes and graphene. Adv. Mater..

[25] Giri P., Chakrabarti P. (2016). Effect of Mg doping in ZnO buffer layer on ZnO thin film devices for electronic applications. Superlattices Microstruct..

[26] Ivetić T.B., Dimitrievska M.R., Finčurb N.L., Đačanin L.R., Gúth I.O., Abramović B.F., Lukić-Petrović S.R. (2014). Effect of annealing temperature on structural and optical properties of Mg-doped ZnO nanoparticles and their photocatalytic efficiency in alprazolam degradation. Ceram. Int..

[27] Iqbal J., Jan T., Ismail M., Ahmad N., Arif A., Khan M., Adil M., Arsha A. (2014). Influence of Mg doping level on morphology, optical, electrical properties and antibacterial activity of ZnO nanostructures. Ceram. Int..

[28] Zeng W., Yang X., Shang M., Xu X., Yang W., Hou H. (2016). Fabrication of Mg-doped ZnO nanofibers with high purities and tailored band gaps. Ceram. Int..

[29] Nishant K., Anchal S. (2018). Green photoluminescence and photoconductivity from screen-printed Mg doped Zn O films. J. Alloys Compd..

[30] Samanta A., Goswami M.N. (2018). Optical properties and enhanced photocatalytic activity of Mg-doped ZnO nanoparticles. Phys. E Low-Dimens. Syst. Nanostruct..

[31] Arshad M., Ansari M.M., Ahmed A.S., Tripathi P., Ashraf S.S.Z., Naqvi A.H., Azam A. (2015). Band gap engineering and enhanced photoluminescence of Mg doped ZnO nanoparticles synthesized by wet chemical route. J. Lumin..

[32] Yang J., Wang Y., Kong J., Yu M., Jin H. (2016). Synthesis of Mg-doped hierarchical ZnO nanostructures via hydrothermal method and their optical properties. J. Alloys Compd..

[33] Wu S., Chen Z., Wang T., Ji X. (2017). A facile approach for the fabrication of Au/ZnO-hollow-sphere-monolayer thin films and their photocatalytic properties. Appl. Surf. Sci..

[34] Aksoy S., Caglar Y., Ilican S. (2012). Sol–gel derived Li–Mg co-doped Zn O films: Preparation and characterization via XRD, XPS, FESEM. J. Alloys Compd..

[35] Chen M., Wang X., Yu Y.H., Pei Z.L., Bai X.D., Sun C., Huang R.F., Wen L.S. (2000). X-ray photoelectron spectroscopy and auger electron spectroscopy studies of A1- doped ZnO films. Appl. Surf. Sci..

[36] Manzhia P., Kumarib R., Alamb M.B. (2018). Mg-doped ZnO nanostructures for efficient Organic Light Emitting Diode. Vacuum.

[37] Gong M., Li Y., Guo Y., Lv X., Dou X. (2018). 2D TiO_2_ nanosheets for ultrasensitive humidity sensing application Benefited by abundant surface oxygen vacancy defects. Sens. Actuators B Chem..

[38] Yu X.X., Wu Y., Dong B., Dong Z.F., Yang X. (2015). Enhanced solar light photocatalytic properties of ZnO nanocrystals by Mg-doping via polyacrylamide polymer method. J. Photochem. Photobiol. A Chem..

[39] Lu Z., Gong Y., Li X., Zhang Y. (2017). MoS_2_-modified ZnO quantum dots nanocomposite: Synthesis and ultrafast humidity response. Appl. Surf. Sci..

[40] Hsu N.F., Chang M., Hsu K.T. (2014). Rapid synthesis of ZnO dandelion-like nanostructures and their applications in humidity sensing and photocatalysis. Mater. Sci. Semicond. Process..

[41] Brouri T., Lescop B., Elies P., Rioual S. (2015). Interplay effects of humidity and UV light sensitivities of Zn0.9Mg0.1O nanogranular thin film. Appl. Surf. Sci..

[42] Kannan P.K., Saraswathi R., Rayappan J.B.B. (2010). A highly sensitive humidity sensor based on DC reactive magnetron sputtered zinc oxide thin film. Sens. Actuators A.

